# *S*urface *Me*asure *t*o *D*epth (SMeTD): a new low-budget system for 3D water temperature measurements for combining with UAV-based thermal infrared imagery

**DOI:** 10.1007/s10661-023-12127-3

**Published:** 2023-11-27

**Authors:** Eva Loerke, Ina Pohle, David Drummond, Pauline E. Miller, Josie Geris

**Affiliations:** 1 University of Aberdeen, Aberdeen, UK; 2 Potsdam, Germany; 3 James Hutton Institute, Aberdeen, UK; 4 Joint Nature Conservation Committee, Aberdeen, UK

**Keywords:** 3D water temperature, Ground truthing, Low-cost sensing, Remote sensing

## Abstract

Characterising spatial patterns in water temperature is important for monitoring aquatic habitats and understanding physical and biogeochemical processes to support environmental management decisions. As freshwater bodies exhibit high spatial and temporal variability, high-resolution 3D temperature data are essential to understand local anomalies. The acquisition of simultaneously high spatial and temporal datasets in the field has so far been limited by costs and/or workload associated with commonly used monitoring systems.

We present a new, low-cost, spatially and temporally flexible 3D water temperature monitoring system, Surface Measures to Depth (SMeTD). SMeTD can be used to provide information on the relation of water surface temperature to changes with depth, characterise water temperature in 3D and ground truth remotely sensed thermal infrared data. The systems performance was tested under laboratory conditions and under controlled conditions in the field. This revealed an accuracy comparable to established but more expensive monitoring systems. Field testing of SMeTD involved 1-min data collection of 3D water temperature for a full diurnal cycle in a lake. The 3D temperature patterns were supported by a thermal infrared image of the lakes surface. The field dataset demonstrated higher water temperatures and higher water temperature variation at the surface compared to deeper layers. SMeTD can be used to observe a broad range of hydrological processes in natural and artificial aquatic environments and help to understand processes involved with energy budgets, infiltration, limnology, or groundwater surface water exchange.

**Supplementary information:**

The online version contains supplementary material available at 10.1007/s10661-023-12127-3.

## Introduction

Water temperature (T_w_) is vital for aquatic habitats and the wider ecosystem (Jackson et al., 2021). It influences physical and biogeochemical processes, thus being one of the main controlling factors for water quality (e.g. Delpla et al., 2009). T_w_ is of economic importance, e.g. for the fishing industry (Donnelly et al., 2020) and the supply of cooling water.

Influenced by natural and anthropogenic factors, T_w_ of freshwater bodies can exhibit wide spatial and temporal variability (see, e.g. review by Somers et al. (2013)). High-resolution 3D T_w_ data are essential for identifying and understanding local anomalies, groundwater upwelling, infiltration and mixing in general (Mejia et al., 2020; Selker et al., 2006). Such understanding is required to inform environmental management decisions, especially in a warming climate (Fullerton et al., 2018).

A wide range of different methods has been applied to measure T_w_ in the field. Single-point sensors with data loggers, such as the *Onset Hobo Tidbit* or the *Gemini Tinytag Aquatic 2*, which offer an accuracy of ± 0.2 °C and ± 0.5 °C, respectively, are commonly used to measure T_w_ at a fixed location with a high temporal resolution. A dense network of several loggers may be applied to also characterise spatial variability of T_w_ (Selker et al., 2006). Fibre-optic distributed temperature sensing (FO-DTS) offers a higher spatial resolution approach but is in general associated with high costs and the need of continuous calibration during data collection to reach accuracies of 0.1 °C (Hare et al., 2015; van de Giesen et al., 2012). Hence, studies such as Dormuth and Leboldus (2011) applied a single array of low-cost temperature loggers (Maxim iButton, accuracy ± 0.5 °C) to measure vertical T_w_ profiles in a stormwater detention pond. We are not aware of any study combining several low-cost temperature logger arrays to analyse 3D T_w_ patterns.

Remotely sensed thermal infrared (TIR) imagery, obtained by satellite, airplane/helicopter or unmanned aerial vehicles (UAV), is seen as a suitable low-cost solution to measure T_w_ at a high spatial resolution. Spatial temperature patterns at the water surface revealed by TIR imagery from UAVs have been used, for example, to identify thermal refuges in rivers (Fakhari et al., 2022) and detect groundwater upwelling (Casas-Mulet et al., 2020; Hare et al., 2015). To obtain absolute temperature values, TIR data require ground truthing which is often achieved by implementing in situ measurements in form of point measurements (Dyba et al., 2022; Lewandowski et al., 2013) and in some cases of FO-DTS (Hare et al., 2015; Marruedo Arricibita et al., 2018). As remotely sensed data are limited to measurements at the surface, which can be highly influenced by the diurnal cycle of net radiation and prevailing weather conditions (Marruedo Arricibita et al., 2018), it is crucial to choose the right location at the water surface for ground truthing measurements. As surface water temperatures are highly affected by ambient conditions (Lewandowski et al., 2013), there are limits to relying on TIR imagery which only measures T_w_ emitted from the upper 0.1 mm of a water body (Torgersen et al., 2001) to understanding 3D T_w_ patterns. T_w_ stratification is characteristic for deep reservoirs (Fukushima et al., 2022), but has also been observed in shallow lakes and rivers (Mejia et al., 2020; Toffolon et al., 2022), despite these often being assumed as well mixed. Hence, because remotely sensed data alone are not capable of capturing 3D spatial thermal variability, combining these with FO-DTS offers a suitable solution (Hare et al., 2015), but again this involves high pressures on resources (costs and labour intensive).

The aim of this study was to develop, test and present a novel low-cost, spatially and temporally flexible 3D water temperature monitoring system, which can be used to (1) ground truth remotely sensed TIR data and (2) can provide information on the relation of surface T_w_ to changes with depth and thereby allow to analyse T_w_ in 3D.

## SMeTD development

### SMeTD hardware

**Fig. 1:**
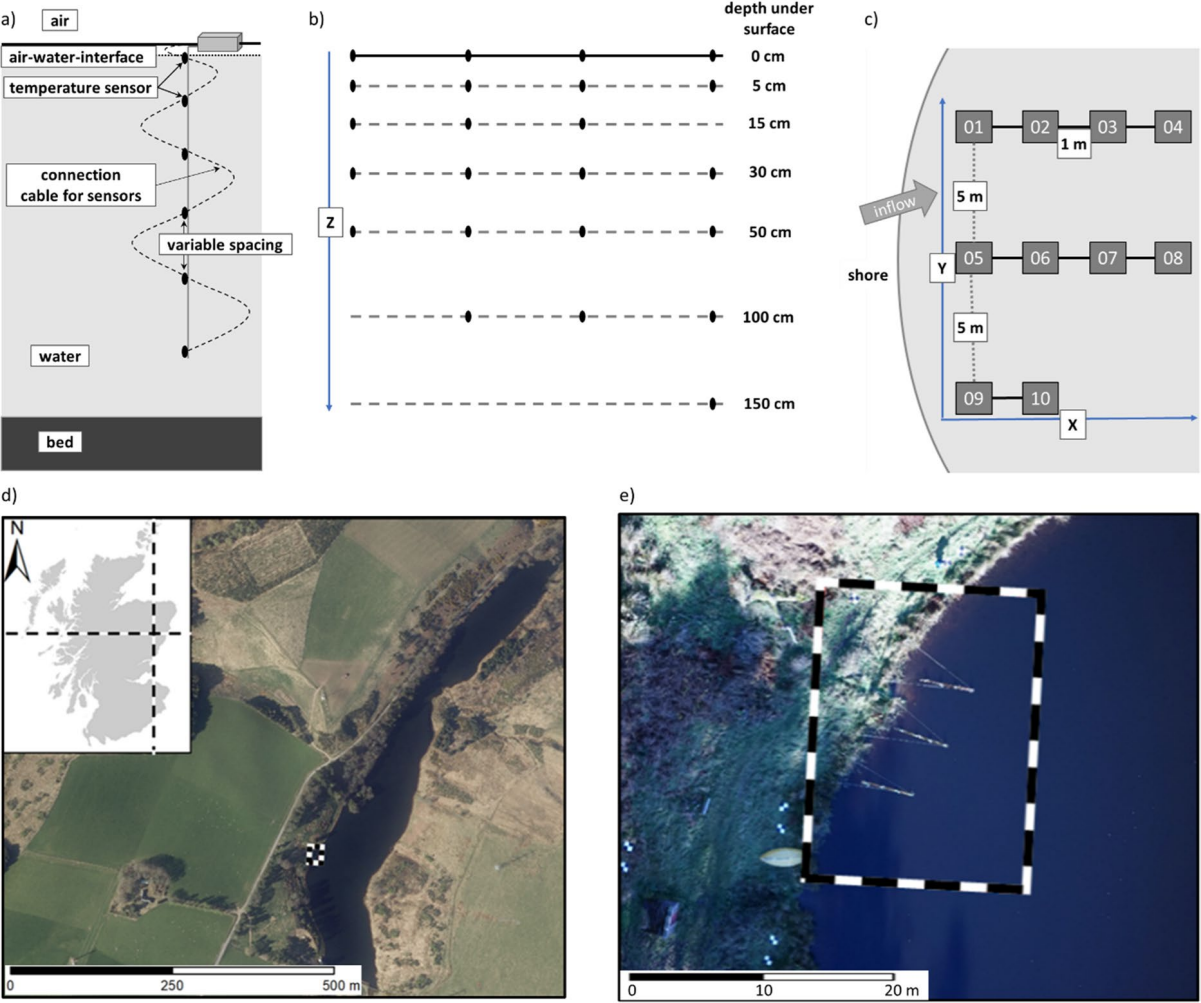
Design idea of **a**) single SMeTD and **b**) a cross-section of SMeTDs arranged in a line and **c**) as a grid. Furthermore, **d**) overview of study site and **e**) setup for field application

Our system is built of several sensor lines which can measure T_w_ at high spatial and temporal resolution (Fig. 1). One sensor line will be hereafter referred to as SMeTD. Each SMeTD has several temperature sensors (MCP9808) which can be placed at flexible depths. MCP9808s are commonly used in a variety of applications including monitoring temperatures in personal computers or servers and offer a typical accuracy of ± 0.25 °C with a user selectable resolution of up to + 0.0625 °C. The total number of sensors and spacing along the cable could be adapted, but for our prototype, six sensors were installed in blanking plugs placed along a 3-m reinforced cable with clips to allow adjustable spacing for flexibility in the spacing with depth (Fig. 1a). Each SMeTD also involves a small ABS (acrylonitrile butadiene styrene) box, containing a microcontroller, battery, real-time clock and SD-card reader. The ABS box can be installed on top of any type of floating device. Several SMeTDs can be arranged in a grid for 3D analysis (Fig. 1b and c).

The detailed circuit diagram for a SMeTD, all parts and a breakdown of costs can be found in the supplementary material S1.

### SMeTD software

The SMeTD program is written on Arduino IDE. It is provided in the supplementary material S1 and can be accessed via GitHub (https://github.com/eva-c-l/SMeTD.git). Once a SMeTD has been started, it creates a new consecutively numbered file on an SD card containing an individual serial number. After the start-up, the program initially powers up all sensors and validates their existence before setting the sensors on “sleep” mode. For one measurement, each sensor is powered up, and a reading is taken and stored and then powered down again before the next sensor is read. The measuring interval can be chosen as multiples of approx. 4 s, excluding inherent time delays in the program and a 500 ms defined delay allowing sensors to settle before taking readings. The readings are saved with a date-and-time stamp provided by an internal real-time clock.

### SMeTD performance

Prior to collecting data in the field, the performance of the sensors was tested against a *Solinst 3001 Levelogger*-—10 m (accuracy: ± 0.05 °C) under different ambient temperatures (~ 4 °C and ~ 19 °C) in a controlled lab experiment (see supplementary material 1).

## Field application of SMeTD

### Field data collection and analysis

The suitability of the SMeTDs to ground truth UAV-based TIR measurements and to analysis T_w_ in 3D was tested at the edge of a lake in northeast Scotland (Fig. 1d). The lake has a maximum depth of 8 m, is approximately 750 m long, 100 m wide and covers an area of approximately 5 ha (see supplementary material S2 for details). The lake represents a relatively uncomplicated first field application, as opposed to a stream where water typically moves more rapidly and which could require additional considerations for data processing.

Ten SMeTDs (i.e. 60 sensors in total) were installed in a grid spanning an area of 10 m width and 6 m off the shore into the lake (Fig. 1d and e). This section of the lake was chosen as it is close to a small natural inflow of water with relatively warm temperature, so that variations in temperature were likely to be observed. T_w_ was measured within the lake for over 27 h at a 1-min interval (start at 12:30 5th November 2020). The ABS box of each SMeTD was installed on a floating platform constructed out of pipe insulation, two times four SMeTDs were arranged as a line using a 4-m-long tent pole, and another line consisted of two SMeTDs. The top sensor of each SMeTD was clipped to the tent poles just underneath the water surface, with approximately 10-cm distance to the ABS box to avoid interference of the ABS box. Welding rods with a length of 70 cm, 100 cm and 150 cm were installed next to the ABS boxes to allow the remaining temperature sensors to be clipped at predefined depths following the profile of the lakebed (Fig. 1b).

A simple moving average of five readings was applied to the SMeTD’s 1-min field dataset. T_a_, wind direction, wind speed and precipitation were monitored at an hourly interval by an automated weather station about 1.5 km northwest of the lake.

A TIR image was collected on 6th November 2020 at 12:06 BST to demonstrate the use of the SMeTD gridded data to ground truth the TIR imagery. The UAV used to gather TIR imagery for this survey was a DJI Matrice 600 Pro, equipped with a standard Sony α7R and an Optris PI450 (thermal infrared camera). The Optris PI450 captures pictures within the near-infrared spectrum (800 nm–1400 nm), with an optical resolution of 382 pixels × 288 pixels and a sensitivity of 40 mK. Prior to the flight, the Optris PI450 was calibrated under laboratory conditions for temperatures 5 to 30 °C using a black body (ISDC’s IR-2103/301). The UAV was programmed to hover at 80 m over the SMeTD grid in the lake, so that the resulting TIR image covered an area of approx. 49 m × 52 m with a resolution of 13.5 cm. The lake part of the TIR image was extracted based on a simultaneously acquired RGB image. Measurements of the surface sensors of the SMeTD grid at 12:06 BST 6th November 2020 were used to ground truth the TIR image. Based on the data, a linear trendline was calculated and then used to calibrate the acquired TIR image. After removing the pixels covering the ABS boxes in the TIR image, the area covered by the SMeTD grid was extracted from the calibrated image.

The calibrated TIR data were used in combination with all the SMeTD’s corresponding T_w_ readings to interpolate a dense point cloud in 3D using the kriging function of the package gstat (Pebesma & Graeler, 2004) in R. While the accuracy of the final 3D data depends on the resolution of the TIR and SMeTD data collected, the visual plots were created using a 2.5 cm × 2.5 cm × 2.5 cm dense point data cloud.

### Field application results

The field SMeTD datasets provide information on temperature changes from the surface to deeper layers of the lake for a complete diurnal cycle. This revealed a delayed response of T_w_ to T_a_ and a clear difference between night- and daytime (supplementary material S3). The spatiotemporal patterns for T_w_ were similar for all SMeTDs, but the magnitude of the amplitude varied depending on the location of the SMeTD. T_w_ variations with depth were very small during the night and increased with the start of the day reaching a maximum variation around noon. The temperature sensors at the surface showed the largest range in temperature; the deepest sensors showed the smallest variations in temperature.

In relation to ground truthing the TIR data, the offset between the surface T_w_ of a SMeTD and the value of the corresponding TIR pixel varied between 3.02 and 4.26 °C. There was a strong linear correlation of the data with a Pearson correlation R^2^ of 0.942 and a *p*-value of < 0.001. As the slope of the linear trendline was 1.06, the TIR image could be corrected by using only the offset value (4.2 °C).

**Fig. 2:**
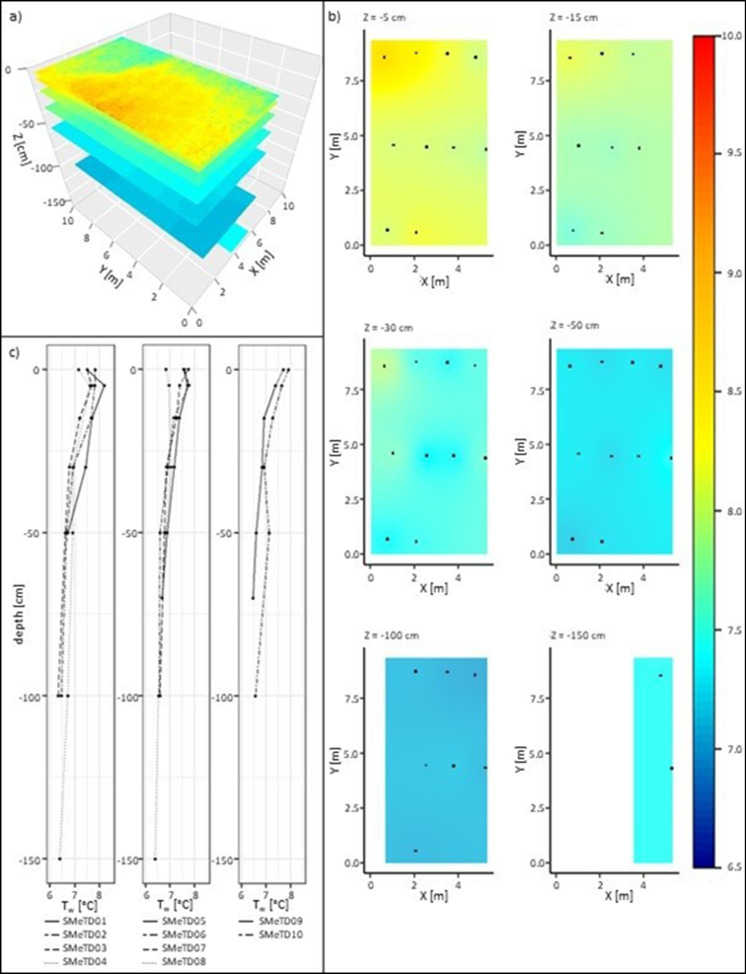
Interpolated T_w_ data. **a** X–Y cross-sections in 3D. **b** X–Y cross-section with locations of SMeTDs (indicated in black). **c** Temperature-depth profiles for individual SMeTDs at time of flight

The interpolated 3D data reflected the combined patterns of the SMeTDs (Fig. 2c) and calibrated TIR image. Together, they revealed higher mean T_w_ and higher variation of T_w_ related to the inflows contribution of warmer water directly at the surface, temperatures ranging between 7.00 and 9.83 °C (Fig. 2a). In agreement with the SMeTD data showing decreasing T_w_ with depth, the spatial variation in T_w_ decreased with depth, and the interpolated data shows a general decrease from an average T_w_ of 8.23 °C at the surface to an average T_w_ 7.41 °C at 150 cm depth (Fig. 2b). The interpolated data reveal horizontal thermal stratification; generally, colder layers were found at depth of 15 cm, 30 cm, 50 cm and 100 cm (Fig. 2a & b).

## Discussion

With a typical accuracy of ± 0.25 °C, the developed sensor system (SMeTD) offers a comparable accuracy to commonly used but more expensive and labour-intensive monitoring systems, such as the FO-DTS or other low-cost data loggers like *Onset Tidbit* or *Gemini Tinytag Aquatic 2*. In an initial test under laboratory conditions (T_a_ ~ 19 °C), the battery used allowed 11,903 readings being taken over a set period of 11,400 min. The maximum battery lifetime under field conditions remains to be tested. The spatial resolution of the SMeTDs is limited by the housing of each individual sensor (in our case 2.5 cm) and comparable to what has been achieved by Hare et al. (2015) who wrapped the cables for FO-DTS around PVC pipes. The maximum length of the cable is limited to a capacity of 400 pF (I2C communication applied). The temporal resolution of the SMeTDs is comparable to temporal resolutions for FO-DTS, which offer fractions of a minute (Selker et al., 2006). In theory, the temporal resolution of SMeTDs could be adjusted to be comparable to Gemini Tinytag Aquatic 2 or Onset Tidbit, both offering temporal resolution of 1 s, but this has not been tested yet. The total costs (applicable in 2022) for our network consisting of 10 SMeTDs (60 sensors) resulted in a fraction of costs (1/6 to 1/50, respectively) that would have been involved building a network out of 60 single other ‘low-cost’ data loggers (£100–£200/item; applicable in 2023) or a FO-DTS (US $50,000 for a full system (Wolf et al., 2023)).

The SMeTD system has been successfully tested in a lake for a full diurnal cycle. While T_w_ appears to be relatively homogenous during the night, the data revealed thermal stratification within the first metre during the day. The influence of ambient conditions like T_a_ and solar radiation is strongest during the day directly at the air–water interface and reduce with depth. These findings correspond with Dormuth and Leboldus (2011) describing thermal stratification in a stormwater detention pond. The thermal pattern at SMeTD01 revealing slightly cooler T_w_ at the surface compared to 5 cm below the surface can be explained by the small natural inflow of warmer water. SMeTD01 was located about a metre north but within close proximity of the inflow.

The surface sensors of the SMeTDs allowed to ground truth the simultaneously acquired TIR imagery without the need of atmospheric or emissivity corrections. Most other studies mostly rely on no or only one logger for correction of a single TIR image (e.g. Casas-Mulet et al., 2020). Here, the 10 sensors used to ground truth one TIR image showed each a slightly different offset, indicating values for corrected TIR images will vary depending on which single sensor is used. Compared to the final offset used (4.2 °C), having only one sensor could lead up to over 1.0 °C of additional uncertainty. The final offset used is within the range observed in other studies (e.g. Hare et al. (2015)).

The combination of SMeTD data and TIR image successfully provided an interpolated 3D T_w_ dataset. While the data showed a clear decrease in T_w_ with depth down to 1 m, there was a small increase in T_w_ from 1 to 1.5 m possibly related to groundwater or surface inflow. The surface T_w_ pattern, which could be related to the inflow and other influences from the shore, is only visible in the TIR imagery and the surface layer of the 3D interpolation; it is only vaguely detectable at the 5-cm depth layer. This may be due to radiation and heat flux influences at the surface (Toffolon et al., 2022) and confirms the findings by Marruedo Arricibita et al. (2018) that the water surface-atmosphere interface had low spatial correlation to lower layers during daytime.

For the SMeTD prototype presented here, the system was limited to a maximum depth of 3 m and six sensors with a maximum spacing of 50 cm. In future development, it could be adapted to accommodate more sensors with a shorter or wider spacing to fit the needs of other applications. As such, the spacing between individual SMeTDs could also be changed to distribute the chains more evenly across a larger area to analyse T_w_ patterns related to processes in lakes in 3D at a larger scale. While the costs mostly depend on the number of sensors per SMeTD, the design itself makes it an affordable and highly versatile system. Depending on the aim of future studies, SMeTD could be applied to observe a broad range of hydrological processes in natural and artificial aquatic environments. It could be applied to understand overall energy budgets, infiltration, limnology, groundwater surface water exchange or similar processes, and the 3D profile could be used for modelling hydrological-free or forced convection.

## Conclusion

The presented novel low-cost water temperature (T_w_) monitoring system SMeTD can be used to analyse T_w_ at high spatial and temporal resolution. SMeTDs can be used to ground truth remote-sensed TIR data and analyse the relation of surface T_w_ to changes with depth allowing 3D T_w_ modelling. While up to date a wide range of different methods have been applied to measure T_w_ in the field, SMeTD bridges the gap of affordability vs high temporal and spatial resolution. The SMeTDs offer a comparable absolute accuracy to other state of the art but more expensive monitoring systems. Due to its simple design and flexibility, SMeTDs can be used to locate thermal anomalies (i.e. thermal refugia) and gain better understanding of hydrological processes such as groundwater upwelling, infiltration and mixing in general.

### Supplementary Information

Below is the link to the electronic supplementary material.

Supplementary file1 (PDF 458 KB)

Supplementary file2 (PDF 261 KB)

Supplementary file3 (PDF 445 KB)

## Data Availability

The data used in this study can be requested from the corresponding author.

## Declarations

### Disclaimer

All of the material is owned by the authors and/or no permissions are required.
