# Supplementary material for: Surface Measure to Depth (SMeTD): a new low-budget system for 3D water temperature measurements for combining with UAV-based thermal infrared imagery
Source: Environ Monit Assess. 2023 Nov 27;195(12):1533. doi: 10.1007/s10661-023-12127-3 (PMC10678821; doi:10.1007/s10661-023-12127-3)
Supplement: Supplementary file 1 — Supplementary file1 (PDF 458 KB) [file 10661_2023_12127_MOESM1_ESM.pdf]

SMeTD
Supplementary Materials 1: SMeTD Specifications

S1.1. Sensor performance

The performance of all individual sensors of all SMeTDs were independently tetsed. First, we tested the performance at different ambient temperatures against a Solinst 3001 Levelogger – 10 m (accuracy: ± 0.05 °C) for each individual sensor of the SMeTDs. A tank filled with 40 L of water was placed in a temperature-controlled cold room (ambient temperature ~ 4 °C) and a temperature-controlled laboratory (ambient temperature ~ 19 °C) respectively. After allowing the water to adjust to ambient conditions the sensors of a SMeTD were installed in the centre of the water tank (Figure S1.1). In addition, we also included a Solinst 3001 Levelogger for independent measurements. The SMeTD and Solinst 3001 Levelogger were set to record T_w with 1 min intervals for approx. 24 hours in the cold room or laboratory.

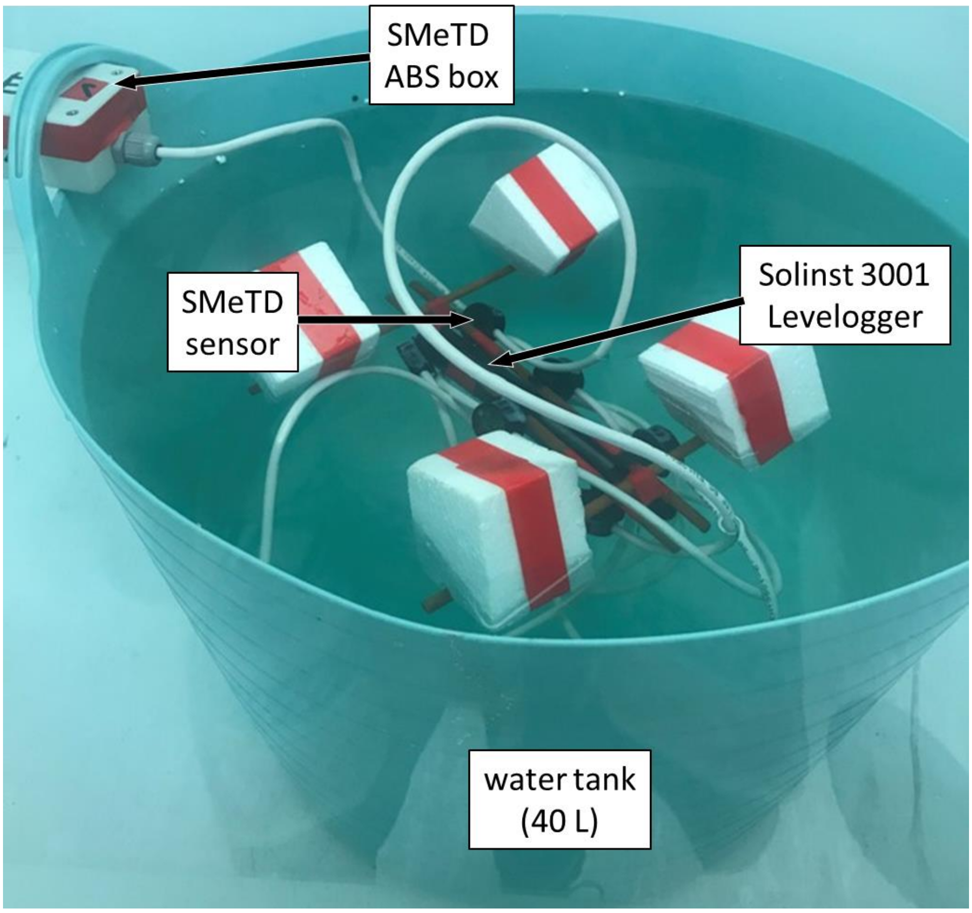

Figure S1.1: Setup for validation of SMeTD under laboratory conditions.

The resulting datasets were used to plot measured T_w of each individual sensor against measured T_w of the Solinst 3001 Levelogger (black diamond symbols in Figure S1.2) R. The first and last 30 min of each dataset were excluded to remove artefacts related to the setup and removal of the sensors. A linear trendline was fitted through the resulting scatterplot and the offset of the trendline with a precision of 2 digits was extracted for each individual sensor. This procedure was repeated for each SMeTD individually. Offset, bias and root mean square error (RMSE) for each individual sensor are summarised in Table S1.1.

Table S1.1: performance test results for individual sensors

SMeTD	sensor	Slope (trendline)	Intercept (trendline)	BIAS	RMSE [°C]
01	1	1.01	0.94	1.02	1.03
	2	1.01	0.48	0.56	0.56
	3	1.01	0.44	0.58	0.59
	4	1.01	0.48	0.61	0.62
	5	1.01	0.61	0.76	0.77
02	1	1.01	0.82	1.04	1.04
	2	1.00	0.62	0.66	0.66
	3	1.01	0.32	0.47	0.47
	4	1.01	0.49	0.66	0.66
	5	1.01	0.58	0.75	0.75
	6	1.00	0.71	0.76	0.76
03	1	1.01	0.86	0.93	0.93
	2	1.00	0.51	0.52	0.53
	3	1.00	0.60	0.60	0.60
	4	1.01	0.58	0.68	0.68
	5	1.00	0.70	0.72	0.73
	6	1.00	0.79	0.83	0.83
04	1	1.01	0.84	0.95	0.95
	2	1.01	0.23	0.37	0.38
	3	1.01	0.52	0.62	0.62
	4	1.01	0.36	0.46	0.47
	5	1.01	0.37	0.49	0.50
	6	1.00	0.73	0.77	0.78
05	1	1.01	0.90	0.77	1.00
	2	1.02	0.14	0.31	0.75
	3	1.01	0.44	0.40	0.55
	4	1.01	0.40	0.53	0.66
	5	1.00	0.64	0.47	0.71
	6	1.01	0.63	0.63	0.89
06	1	1.01	0.90	1.00	1.00
	2	1.02	0.14	0.33	0.35

06	3	1.01	0.44	0.58	0.59
	4	1.01	0.40	0.51	0.51
	5	1.00	0.64	0.63	0.63
	6	1.01	0.63	0.74	0.74
07	1	1.01	0.75	0.90	0.90
	2	1.01	0.44	0.51	0.51
	3	1.01	0.45	0.57	0.58
	4	1.01	0.50	0.60	0.61
	5	1.01	0.55	0.64	0.65
	6	1.01	0.62	0.68	0.69
08	1	1.01	0.89	1.00	1.00
	2	1.00	0.75	0.75	0.75
	3	1.00	0.52	0.55	0.55
	4	1.01	0.59	0.66	0.66
	5	1.01	0.62	0.71	0.71
	6	1.00	0.88	0.89	0.89
09	1	1.01	0.95	1.07	1.07
	2	1.00	0.56	0.62	0.63
	3	1.00	0.59	0.63	0.63
	4	1.01	0.53	0.63	0.63
	5	1.01	0.61	0.74	0.74
	6	1.00	0.78	0.82	0.82
10	1	1.00	0.95	0.97	0.97
	2	1.01	0.55	0.61	0.61
	3	1.01	0.35	0.46	0.47
	4	1.01	0.50	0.60	0.61
	5	1.01	0.16	0.30	0.32
	6	1.01	0.59	0.69	0.69

Second, to test the linear correlation between sensors and the Solinst 3001 Levelogger the experiment was then repeated under controlled field conditions. This involved an identical setup to that in the cold room and laboratory, but outside (shielded against direct solar radiation) under natural air temperature variations in Aberdeen, Scotland, UK for a complete diurnal cycle. T_w during this experiment varied between 10 °C and 15 °C (red cross symbols in Figure S1.2) and is thus within the temperature range of the 2-point validation. The validation dataset from the controlled field experiment (SMeTD 10) confirmed the suitability of the 2-point validation with R^2 ranging between 0.935 and 0.998 for all sensors of SMeTD 10. As an example, Figure S1.2 shows the linear response of both validation datasets for SMeTD 10 sensor 6.

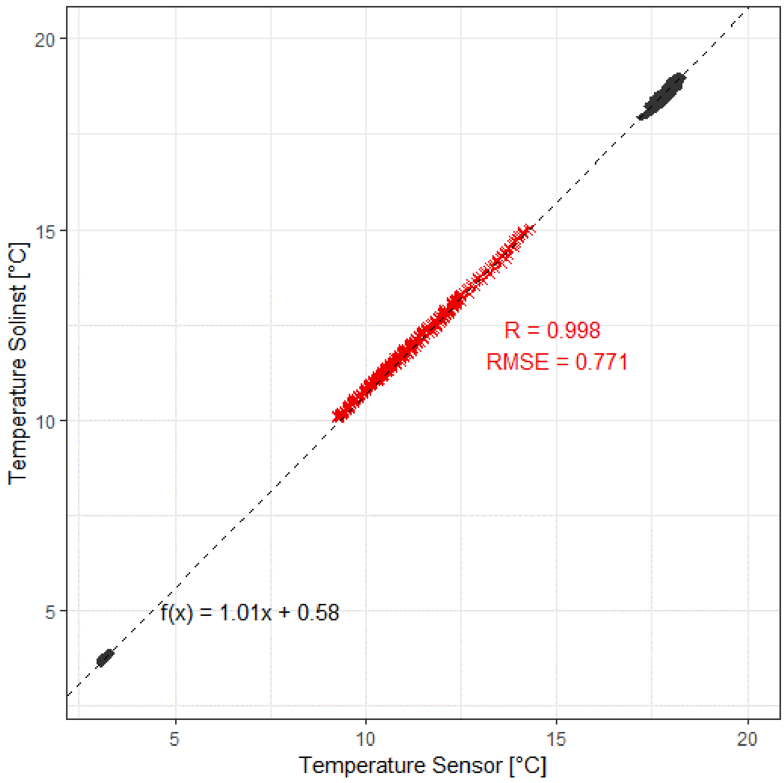

Figure S1.2: scatterplot of data from performance test for SMeTD 10 - sensor 6.

Bias and RMSE for each sensor were calculated using the Metrics package (Hamner et al., 2018) in R. The average RMSE for each SMeTD ranged between 0.61 and 0.76 (Table S1.2) and is acceptable considering the setup of the validation experiments as well as the accuracies of the sensors and the *Solinst 3001 levelogger*.

Table S1.2: average RMSE for individual SMeTD based on laboratory experiments

SMeTD	average RMSE [°C]
01	0.71
02	0.73
03	0.72
04	0.62
05	0.76
06	0.64
07	0.66
08	0.76
09	0.75
10	0.61

S1.2. Circuit diagram of a SMeTD

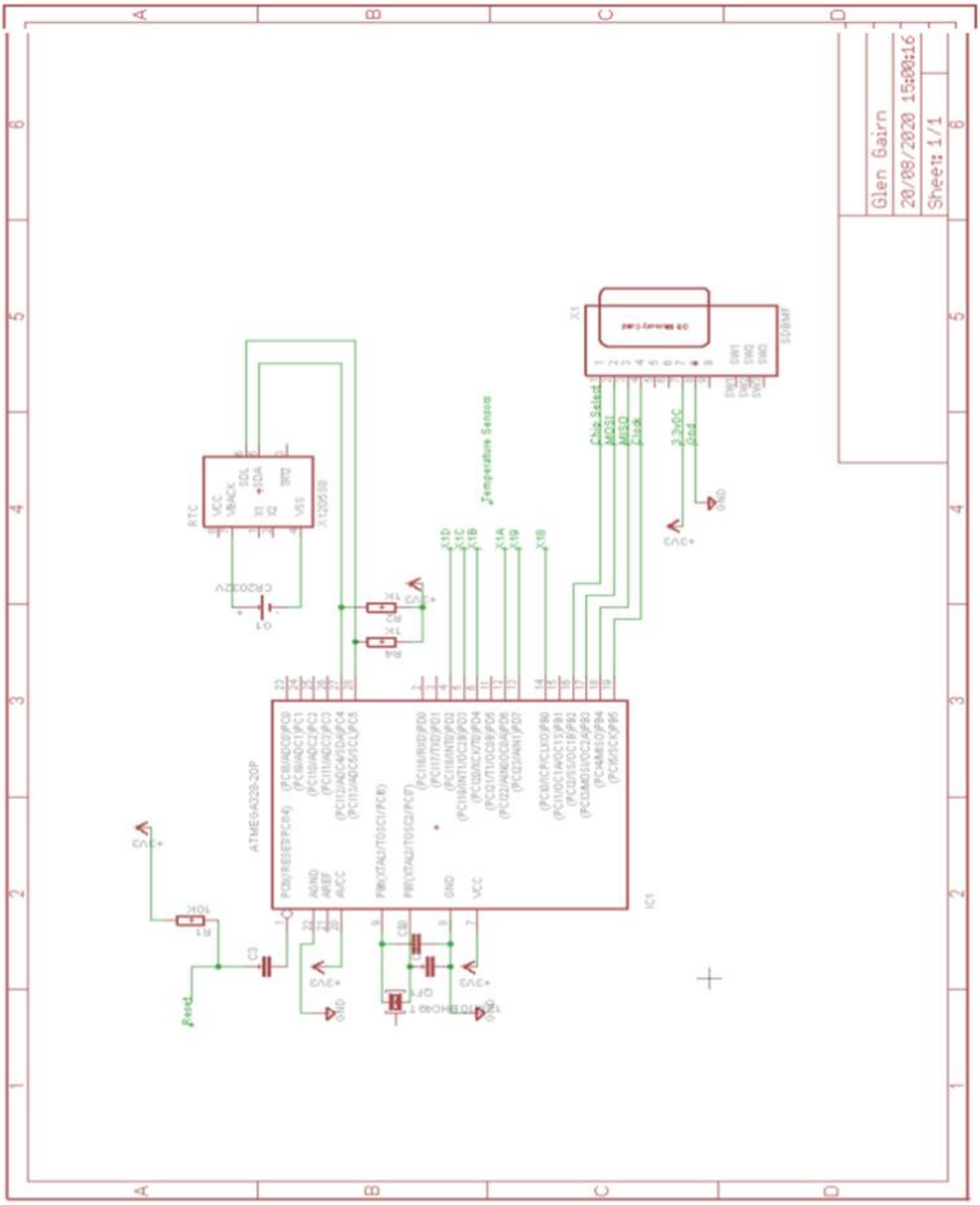

S1.3. Parts and costs for a SMeTD (based on the build of ten SMeTDs in 2020)

part	Qty	Cost each	Total
Small ABS Box	1	£6.91	£6.91
Sensor Adaptor	6	£1.67	£10.02
Capacitor 100nF	7	£0.17	£1.20
Processor Atmega 328P	1	£0.35	£0.35
Crystal	1	£0.32	£0.32
Capacitor 22pF	2	£0.19	£0.38
Cable Gland	1	£0.76	£0.76
Gland lock nut	1	£0.22	£0.22
Electric fence post	1	£1.80	£1.80
Sensor housing	6	£0.06	£0.35
Clip	6	£0.36	£2.14
SD Card adaptor	1	£4.43	£4.43
SD Card (8GB SDHC)	1	£14.12	£14.12
Switch	1	£0.62	£0.62
Resistor 10k	2	£0.01	£0.02
Resistor 1K	2	£0.05	£0.11
Button Battery	1	£0.89	£0.89
High Accuracy Pi RTC I2C Module	1	£6.12	£6.12
DIL socket	1	£0.21	£0.21
Amber Epoxy Potting Compound	0.1	£20.98	£2.10
Microchip MCP9808-E/MS, Temperature Converter	6	£1.07	£6.42
Lithium Polymer Rechargeable Battery	1	£17.88	£17.88
Circuit board	0.1	£12.53	£1.25
Contact Adhesive		£3.28	£3.28
**Total costs**			**£81.89**

S1.4. Arduino Program

```
/***************************************************************************
  This is a program to read six temperature sensors based on the MCP98078
  microchip.  It has been designed for Eva Loerke, a student at The James
  Hutton Institute, and will be used to measure the temperature of water
  at depths of: Surface, 5cm below surface and then further depths of 10cm, 20cm,
  35cm and 55cm. (However, these depths can be amended at any time.) The air temperature
  will also be logged.
  The unit, and its associated software, has been designed and built by
  David Drummond of The James Hutton Institute.
  November 2019
*/

#include <Wire.h>
#include <Adafruit_Sensor.h>
#include "Adafruit_MCP9808.h"
#include <SPI.h>
#include <SD.h>
#include <LowPower.h>
#include "RTClib.h"
#include <avr/wdt.h>
#include <EEPROM.h>



//RTC_DS1307 rtc;
RTC_DS3231 rtc;

char myDateString[10]; //19 digits plus the null char
char myTimeString[10]; //19 digits plus the null char

/*************THE FOLLOWING LINE SETS THE INTERVAL FOR READINGS*****************
   The interval is set multiples of 4 seconds but this can be chaged on the 'SLEEP' tab
   A sleep time of 0 will set the interval to every 4 seconds (approx)
   Note: the exact interval will be different because there are inherent time delays
   in the program as well as programmed delays.
*/
int sleepTime = 13; // a sleep time of 225 gives approx 900 seconds (15 Minutes) of sleep at 4 second intervals.
/******************************************************************************
*/

int sleepCount = 0, ID, num = 0000;
//const int SDPower = 9;
const int chipSelect = 10;  //this is the correct pin when using the GPS Shield.  Any other pin may be used if desired for other setups
int oldDay;

File dataFile;
char filename[] = "00000000.TXT";
float c1, c2, c3, c4, c5, c6;
```

```
Adafruit_MCP9808 tempsensor = Adafruit_MCP9808();
#define sleepNow (true)  //enables the sleep function
#define SDCard (true) //make false if you dont want to save the data
#define TCAADDR 0x70
int newDay = 0; //adds a date stamp to the sd card

void setup() {
  Serial.begin(9600);
  wdt_enable(WDTO_8S);   // Enable the watchdog timer with an 8 second timeout
  while (!Serial); //waits for serial terminal to be open, necessary in newer arduino boards.
  Serial.println(F("The James Hutton Institute Water Temperature Logger"));
  if (!SDCard)Serial.println(F("      ~~~~~~~~~~~PLEASE ENSURE YOU HAVE ENABLED THE SD CARD
FOR THIS SENSOR IF REQURED~~~~~~~~~~~"));
  if (!sleepNow)Serial.println(F("    ~~~~~~~~~~~PLEASE ENSURE YOU HAVE ENABLED THE SLEEP
FUNCTION FOR THIS SENSOR IF REQURED~~~~~~~~~~"));
  Serial.print(F("LOGGER INTERVAL IS SET TO:  ")); Serial.print((sleepTime * 4) / 60); Serial.print(F("
MINUTES")); Serial.print(F(" and ")); Serial.print(sleepTime * 4); Serial.println(F(" seconds"));
  pinMode(2, OUTPUT);  //these line allocate power to to each of the sensors
  pinMode(3, OUTPUT);
  pinMode(4, OUTPUT);
  pinMode(6, OUTPUT);
  pinMode(7, OUTPUT);
  pinMode(8, OUTPUT);
  //pinMode(SDPower, OUTPUT);


/****************************************************************************/
  setRTC();  //Set the time on the RTC if required
  // The following line is used for debugging only.  Comment out for normal use
  //Scan();  //Run the 'I2CScan' tab
  /****************************************************
      The following lines will apply power to each sensor and check to see if it exists, then remove
power
      to save battery life.  This sequence only runs once on power up.
  */
  wdt_reset();  //  If all is well then the watchdog timer will be reset;
  digitalWrite(8, HIGH);  //turn on sensor number 8 which is connected to I2C address 0x18
  delay(200);
  tcaselect(0);
  if (!tempsensor.begin(0x18)) {
    Serial.println(F("Couldn't find MCP9808 #1! (0X18)Check your connections and verify the
address is correct.")); //Air
    //while (1);
  }
  digitalWrite(8, LOW);

  digitalWrite(7, HIGH);
  delay(200);
  tcaselect(1);
  if (!tempsensor.begin(0x19)) {
```

```
    Serial.println(F("Couldn't find MCP9808 #2! (0X19)Check your connections and verify the
address is correct.")); //Top
    //while (1);
  }
  digitalWrite(7, LOW);

  digitalWrite(6, HIGH);
  delay(200);
  tcaselect(2);
  if (!tempsensor.begin(0x1A)) {
    Serial.println(F("Couldn't find MCP9808 #3! (0X1A)Check your connections and verify the
address is correct."));  //1st
    //while (1);
  }
  digitalWrite(6, LOW);

  digitalWrite(4, HIGH);
  delay(200);
  tcaselect(3);
  if (!tempsensor.begin(0x1B)) {
    Serial.println(F("Couldn't find MCP9808 #4! (0X1B)Check your connections and verify the
address is correct."));  //1st
    //while (1);
  }
  digitalWrite(4, LOW);

  digitalWrite(3, HIGH);
  delay(200);
  tcaselect(4);
  if (!tempsensor.begin(0x1C)) {
    Serial.println(F("Couldn't find MCP9808 #5! (0X1C)Check your connections and verify the
address is correct."));  //1st
    //while (1);
  }
  digitalWrite(3, LOW);

  digitalWrite(2, HIGH);
  delay(200);
  tcaselect(5);
  if (!tempsensor.begin(0x1D)) {
    Serial.println(F("Couldn't find MCP9808 #6! (0X1D)Check your connections and verify the
address is correct."));  //1st
    //while (1);
  }
  digitalWrite(2, LOW);

  //  If you have enabled the SD card (line48):
  if (SDCard) {
    //digitalWrite(SDPower, HIGH);
    delay(500);
    Serial.print(F("Initializing SD card..."));
```

```
    // see if the card is present and can be initialized:
    if (!SD.begin(chipSelect)) {
      Serial.println(F("Card failed, or not present"));
    }
    else {
      Serial.println(F("card initialized."));
    }
  }

  tempsensor.setResolution(3); // sets the resolution mode of all sensors, the modes are defined
in the table below:
  // Serial.print(F("Sensor resolution in mode: "));
  // Serial.println (tempsensor.getResolution());
  // Mode Resolution SampleTime
  //  0    0.5°C      30 ms
  //  1    0.25°C     65 ms
  //  2    0.125°C    130 ms
  //  3    0.0625°C   250 ms

  /****************************************************8
     The following procedure allocates a new file on the SD card every time powwer is applied
     This ensures that no data is overwritten
     You can create up to 1999 files
  */
  if (SDCard) {
    EEPROM.get(0, ID);
    Serial.print("Unit serial number is: "); Serial.println(ID);
    sprintf(filename, "JH%02d%03d", ID, num);
    //strcpy(filename, fileName);
    for (uint32_t i = 0; i < 2000; i++) {
      filename[4] = '0' + i / 1000;
      filename[5] = '0' + (i % 1000) / 100;
      filename[6] = '0' + (i % 100) / 10;
      filename[7] = '0' + i % 10;
      // create if does not exist, do not open existing, write, sync after write
      if (! SD.exists(filename)) {
        break;
      }
    }
    dataFile = SD.open(filename, FILE_WRITE);
    if (dataFile ) {
      dataFile.print("Unit Serial Number: "); dataFile.println(ID);
      Serial.print("Data will be written to "); Serial.println(filename);
    }
    else {
      Serial.print(F("Couldnt create ")); Serial.println(filename);
    }
    dataFile.close();
  }
  wdt_reset();  //  If all is well then the watchdog timer will be reset;
```

```
  DateTime now = rtc.now();
  sprintf(myDateString, "%4d-%02d-%02d" , now.year(), now.month(), now.day());
  sprintf(myTimeString, "%02d:%02d:%02d" , now.hour(), now.minute(), now.second());
  delay(100);
  Serial.print("Date is: "); Serial.println(myDateString);
  Serial.print("Time is: "); Serial.println(myTimeString);
}
/*******************************END OF SETUP
PROCEDURE*************************/

/*************** ALLOCATE ADDRESS FOR THE
SENSORS*******************************/
void tcaselect(uint8_t i) {
  if (i > 7) return;

  Wire.beginTransmission(TCAADDR);
  Wire.write(1 << i);
  Wire.endTransmission();
}
/********************************************************************************
*/



void ReadSensors() {
  /***************************************************
     Each sensor is powered up in turn,
     a reading is taken and stored then powered down again
     before the next sensor is read
   **************************************************/
  digitalWrite(8, HIGH);
  int x = 0;
  while (!tempsensor.begin(0x18)) {  //If the sensor didnt wake in time then it is read up to 100
times, this resolved a bug.
    delay(50);
    x++;
    if (x == 100); {
      break;
    }
    Serial.println(F("Couldn't find MCP9808 #1! (0X18)Check your connections and verify the
address is correct."));  //1st
  }
  tempsensor.wake();   // wake up, ready to read!
  delay(100);
  tcaselect(0);
  c1 = tempsensor.readTempC();  // read the sensor value and store it in variable c1
  c1 = c1 - 0.6700;  //calibration factor (different for every sensor).
  //Serial.print(F("Sensor 1(X018): "));
  //Serial.print(c1, 4); Serial.print(F("\t"));
  delay(100);
  digitalWrite(8, LOW);
```

```
/**********************************/

digitalWrite(7, HIGH);
while (!tempsensor.begin(0x19)) {
  delay(50);
  x++;
  if (x == 100); {
    break;
  }
  Serial.println(F("Couldn't find MCP9808 #2! (0X19)Check your connections and verify the
address is correct."));  //1st
}
tempsensor.wake();   // wake up, ready to read!
delay(100);
tcaselect(1);
c2 = tempsensor.readTempC();
c2 = c2 - 0.3400;
//Serial.print(F("Sensor 2(X019): "));
// Serial.print(c2, 4); Serial.print(F("\t"));
delay(100);
digitalWrite(7, LOW);
/*************************************/

digitalWrite(6, HIGH);
while (!tempsensor.begin(0x1A)) {
  delay(50);
  x++;
  if (x == 100); {
    break;
  }
  Serial.println(F("Couldn't find MCP9808 #3! (0X1A)Check your connections and verify the
address is correct."));  //1st
}
tempsensor.wake();   // wake up, ready to read!
delay(100);
tcaselect(3);
c3 = tempsensor.readTempC();
c3 = c3 - 0.2981;
//Serial.print(F("Sensor 3(X01A): "));
//Serial.print(c3, 4); Serial.print(F("\t"));
delay(100);
digitalWrite(6, LOW);
/*************************************/

digitalWrite(4, HIGH);
while (!tempsensor.begin(0x1B)) {
  delay(50);
  x++;
  if (x == 100); {
    break;
  }
```

```
    Serial.println(F("Couldn't find MCP9808 #4! (0X1B)Check your connections and verify the address is correct."));  //1st
  }
  tempsensor.wake();   // wake up, ready to read!
  delay(100);
  tcaselect(4);
  c4 = tempsensor.readTempC();
  c4 = c4 - 0.3391;
  //Serial.print(F("Sensor 4(X01B): "));
  //Serial.print(c4, 4); Serial.print(F("\t"));
  delay(100);
  digitalWrite(4, LOW);
  /**********************************/

  digitalWrite(3, HIGH);
  while (!tempsensor.begin(0x1C)) {
    delay(50);
    x++;
    if (x == 100); {
      break;
    }
    Serial.println(F("Couldn't find MCP9808 #5! (0X1C)Check your connections and verify the address is correct."));  //1st
  }
  tempsensor.wake();   // wake up, ready to read!
  delay(100);
  tcaselect(5);
  c5 = tempsensor.readTempC();
  c5 = c5 - 0.3391;
  //Serial.print(F("Sensor 5(X01C): "));
  // Serial.print(c5, 4); Serial.print(F("\t"));
  delay(100);
  digitalWrite(3, LOW);
  /*********************************/

  digitalWrite(2, HIGH);
  while (!tempsensor.begin(0x1D)) {
    delay(50);
    x++;
    if (x == 100); {
      break;
    }
    Serial.println(F("Couldn't find MCP9808 #6! (0X18D)Check your connections and verify the address is correct."));  //1st
  }
  tempsensor.wake();   // wake up, ready to read!
  delay(100);
  tcaselect(6);
  c6 = tempsensor.readTempC();
  c6 = c6 - 0.4378;
  //Serial.print(F("Sensor 6(X01D): "));
```

```
  //Serial.println(c6, 4); //Serial.println(F("\t"));
  delay(100);
  digitalWrite(2, LOW);
}

void Scan() {
  /*****************************************************
     THE FOLLOWING PROCEDURE WILL POWER UP ALL THE SENSORS
     AND THEN SCAN THE I2C BUS TO DETERMINE WHICH SENSOR
     EXISTS AND WHAT ITS ASSOCIATED ADDERESS IS
     (This is primarily used for debugging purposes)
   *****************************************************/

  Wire.begin();  //Starts the I2C
  byte error, address;
  int nDevices;
  for (int powerPin = 2; powerPin < 9; powerPin++)digitalWrite(powerPin, HIGH); //Applies power
to all sensors
  delay(500); //500ms delay to allow sensors to settle before taking readings
  Serial.println(F("Scanning..."));

  nDevices = 0;
  for (address = 1; address < 127; address++ )
  {
    Wire.beginTransmission(address);
    error = Wire.endTransmission();

    if (error == 0)
    {
      Serial.print(F("I2C device found at address 0x"));
      if (address < 16)
        Serial.print("0");
      Serial.print(address, HEX);
      Serial.println("");

      nDevices++;
    }
    else if (error == 4)
    {
      Serial.print(F("Unknown error at address 0x"));
      if (address < 16)
        Serial.print("0");
      Serial.println(address, HEX);
    }
  }
  if (nDevices == 0)
    Serial.println(F("No I2C devices found\n"));
  else
    Serial.println(F("done\n"));
}
```

```
// Make sure the sensor is found, you can also pass in a different i2c
// address with tempsensor.begin(0x19) for example. Can be left in blank for default address use.
// The following table shows all addresses possible for this sensor, you can connect multiple
sensors
// to the same i2c bus, just configure each sensor with a different address and define multiple
objects for that ~(max 8)
//  A2 A1 A0 address
//  0  0  0   0x18  this is the default address
//  0  0  1   0x19
//  0  1  0   0x1A
//  0  1  1   0x1B
//  1  0  0   0x1C
//  1  0  1   0x1D
//  1  1  0   0x1E
//  1  1  1   0x1F


/*******************************************************************************
*/

void readRTC() {
  DateTime now = rtc.now();
  if (now.day() != oldDay) {
    //Serial.println("It's a new day!!");
    sprintf(myDateString, "%4d-%02d-%02d" , now.year(), now.month(), now.day());
    //digitalWrite(SDPower, HIGH);
    delay(500);
    dataFile = SD.open(filename, FILE_WRITE);
    dataFile.println(myDateString);
    dataFile.close();
    oldDay = now.day();
  }
  sprintf(myTimeString, "%02d:%02d:%02d" , now.hour(), now.minute(), now.second());
  delay(100);
}

void setRTC()
{
  if (! rtc.begin()) {
    Serial.println(F("Couldn't find RTC"));
    while (1);
  }
  DateTime now = rtc.now();
  if (rtc.lostPower()) {
    Serial.println(F("RTC lost power, lets set the time!"));
    Serial.print('\n');
    // following line sets the RTC to the date & time this sketch was compiled
    char compileDate[] PROGMEM = __DATE__; char compileTime[] PROGMEM = __TIME__;
    Serial.print("Compile time =  "); Serial.println(compileDate); Serial.println(compileTime);
    rtc.adjust(DateTime(F(__DATE__), F(__TIME__)));
    // This line sets the RTC with an explicit date & time, for example to set
```

```
    // January 21, 2014 at 3am you would call:
    // rtc.adjust(DateTime(2014, 1, 21, 3, 0, 0));
  }
}

/******************************************************************************
*/
void GoToSleep()
{
  while (sleepCount < sleepTime) {
    sleepCount++;
    wdt_reset();  //  If all is well then the watchdog timer will be reset;
    LowPower.powerDown(SLEEP_4S, ADC_OFF, BOD_OFF);  //Change this to suit program.  The
following table shows all possible values
  }
  sleepCount = 0;
}

/*
  SLEEP_15MS,
  SLEEP_30MS,
  SLEEP_60MS,
  SLEEP_120MS,
  SLEEP_250MS,
  SLEEP_500MS,
  SLEEP_1S,
  SLEEP_2S,
  SLEEP_4S,
  SLEEP_8S,
  SLEEP_FOREVER
*/
/******************************************************************************
*/

void loop() {
  wdt_reset();  //  If all is well then the watchdog timer will be reset;
  readRTC();
  ReadSensors();  //Run 'ReadSensors' tab
  /****************THE FOLLOWING LINES WRITE THE RECORDED DATA TO THE SD
CARD******/
  if (SDCard) {
    //digitalWrite(SDPower, HIGH);
    delay(500);
    dataFile = SD.open(filename, FILE_WRITE);
    if ( ! dataFile ) {
      Serial.print(F("Couldnt create ")); Serial.println(filename);
    }
    Serial.print(F("Writing to ")); Serial.println(filename);
    if (dataFile) {
      dataFile.print(myTimeString); dataFile.print("\t");
      dataFile.print(c1, 4); dataFile.print("\t");
```

```
    dataFile.print(c2, 4); dataFile.print("\t");
    dataFile.print(c3, 4); dataFile.print("\t");
    dataFile.print(c4, 4); dataFile.print("\t");
    dataFile.print(c5, 4); dataFile.print("\t");
    dataFile.println(c6, 4);
    dataFile.close();
   }

   // if the file isn't open, pop up an error:
   else {
    Serial.print(F("error opening ")); Serial.print(filename); Serial.println(F(".  The data is not being saved to the SD card"));
   }
   //digitalWrite(SDPower, LOW); // turn the sd card off
  }
  Serial.print(myTimeString); Serial.print("\t");
  Serial.print(c1, 4); Serial.print("\t");
  Serial.print(c2, 4); Serial.print("\t");
  Serial.print(c3, 4); Serial.print("\t");
  Serial.print(c4, 4); Serial.print("\t");
  Serial.print(c5, 4); Serial.print("\t");
  Serial.println(c6, 4);
  delay(100);
  Serial.flush();

  if (sleepNow) GoToSleep(); //If you have enabled the sleep function (line 47) then run the 'Sleep' tab
}
```

Reference

Hamner, B., Frasco, M., LeDell, E., 2018. Metrics: Evaluation metrics for machine learning. R Packag. version 0.1 4, 2018.
