# Supplementary material for: Surface Measure to Depth (SMeTD): a new low-budget system for 3D water temperature measurements for combining with UAV-based thermal infrared imagery
Source: Environ Monit Assess. 2023 Nov 27;195(12):1533. doi: 10.1007/s10661-023-12127-3 (PMC10678821; doi:10.1007/s10661-023-12127-3)
Supplement: Supplementary file 2 — Supplementary file2 (PDF 261 KB) [file 10661_2023_12127_MOESM2_ESM.pdf]

Supplementary Materials 2: Field site description

The suitability of the SMeTDs to ground-truth UAV based TIR measurements and analyse T_w in 3D was tested in Loch Saugh, a lake at Glensaugh, Northeast Scotland (Figure S2.1). We opted for testing the new monitoring system in a lake and not a stream to avoid possible complications with rapidly moving water but chose a location which includes a small inflow to have some form of temperature variation in the datasets. Loch Saugh is approximately 750 m long, 100 m wide and covers an area of 5 hectares. It reaches its largest depth of 8 m at its southern end, which is formed by a dam.

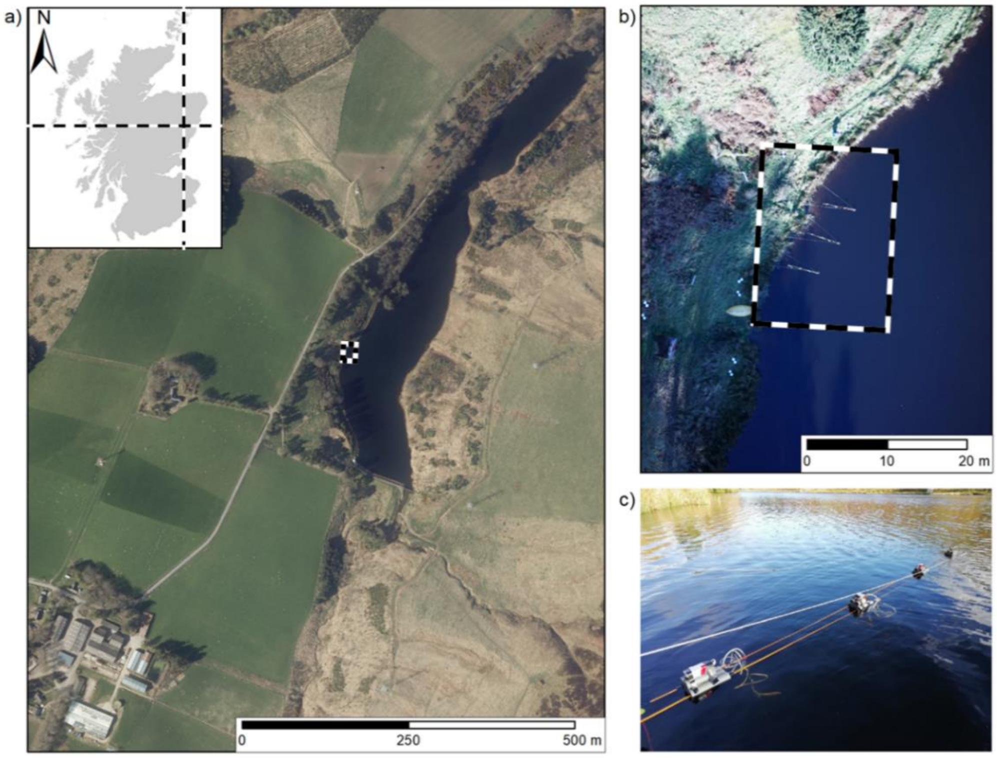

Figure S2.1: a) Location of test site at Loch Saugh (dam marked by yellow dashed line) with b) the area covered in the UAV based TIR-imagery (dashed square) and c) photo of the installation of one SMeTD line in the lake.

The 10 SMeTDs (i.e. 60 sensors in total) were installed in a grid on the west shore of Loch Saugh (Figure S2.1) to measure temperature within the lake for over 27 hours at a 1 min interval (start at 12:30 am 5th November 2020). Air temperature, wind direction, windspeed and precipitation were monitored at an hourly interval by an automated weather station about 1.5 km northwest of the Loch. Over the 27 hours air temperature varied between 8.1 °C and 13.6 °C, windspeed between 0.4 m/s and 3.6 m/s and the total precipitation was 0 mm. At time-of-flight air temperature was 11.1 °C, wind speed 1.3 m/s and wind direction south-west (see supplementary material 3).
