# Supplementary material for: Surface Measure to Depth (SMeTD): a new low-budget system for 3D water temperature measurements for combining with UAV-based thermal infrared imagery
Source: Environ Monit Assess. 2023 Nov 27;195(12):1533. doi: 10.1007/s10661-023-12127-3 (PMC10678821; doi:10.1007/s10661-023-12127-3)
Supplement: Supplementary file 3 — Supplementary file3 (PDF 445 KB) [file 10661_2023_12127_MOESM3_ESM.pdf]

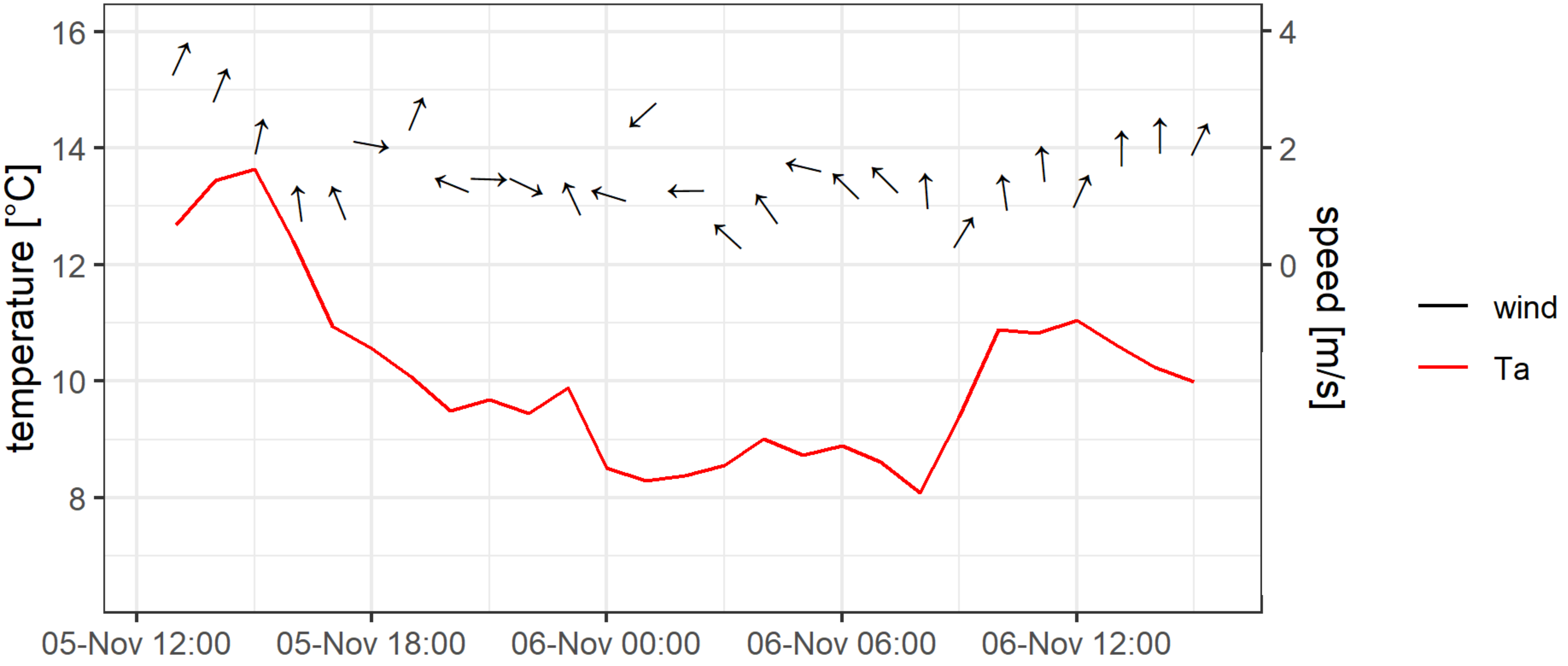

Figure S3.1: ambient conditions during field experiment from nearby weather station. Arrow indicates the wind direction with pointing straight up being true North

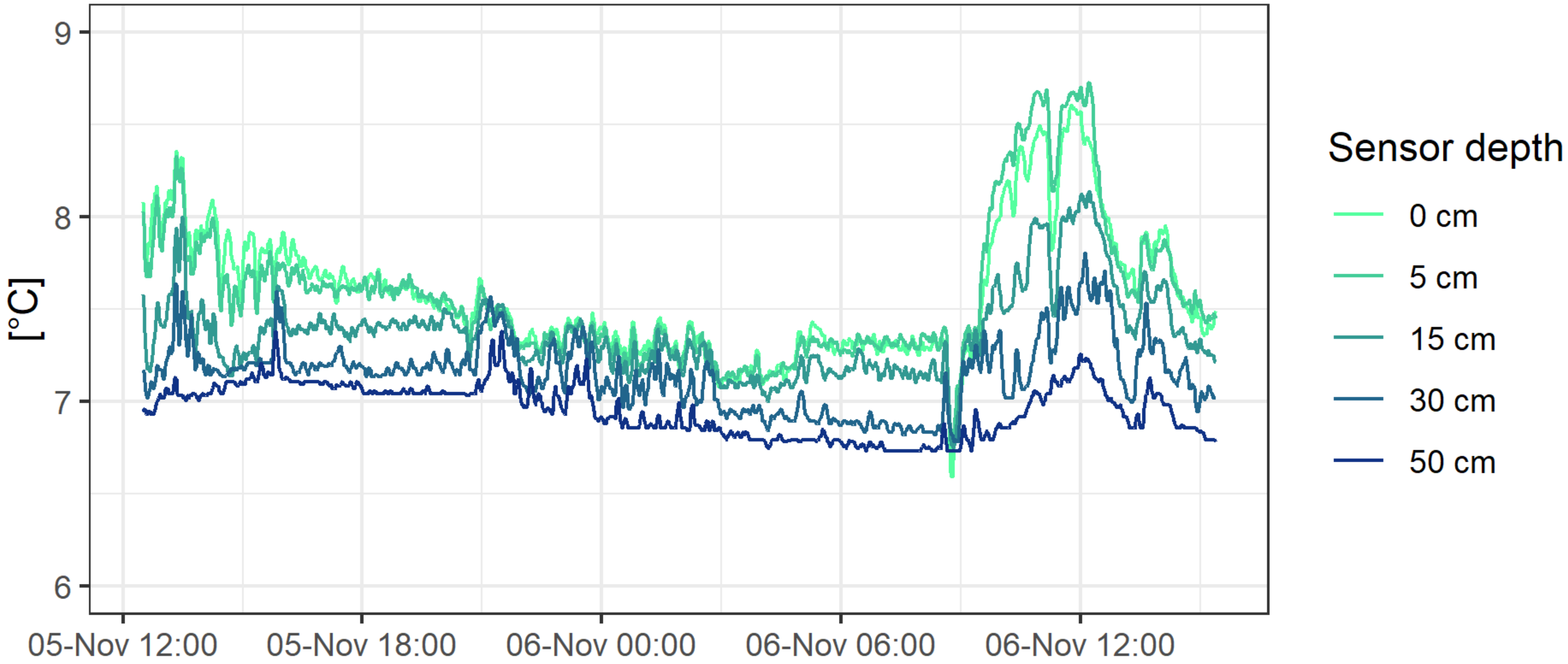

Figure S3.2: complete timeseries for SMeTD01 during field experiment

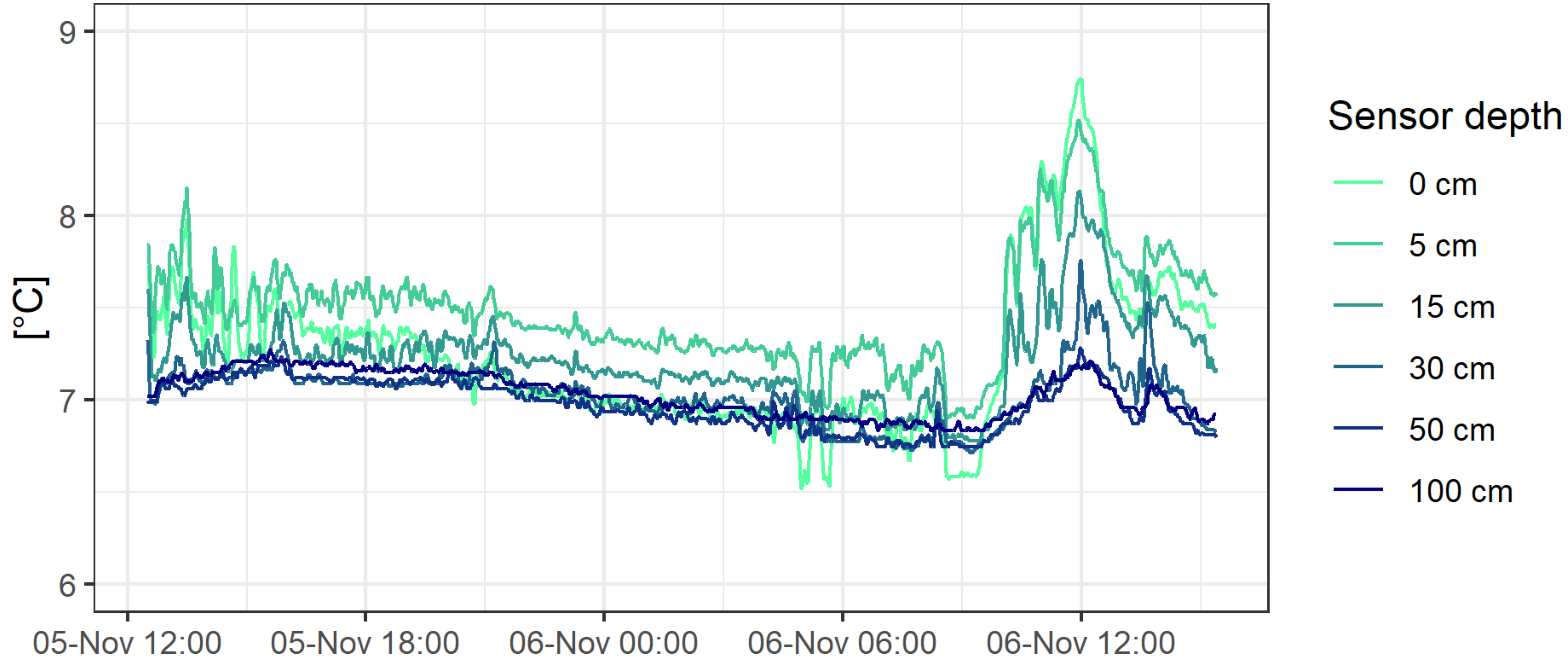

Figure S3.3: complete timeseries for SMeTD02 during field experiment

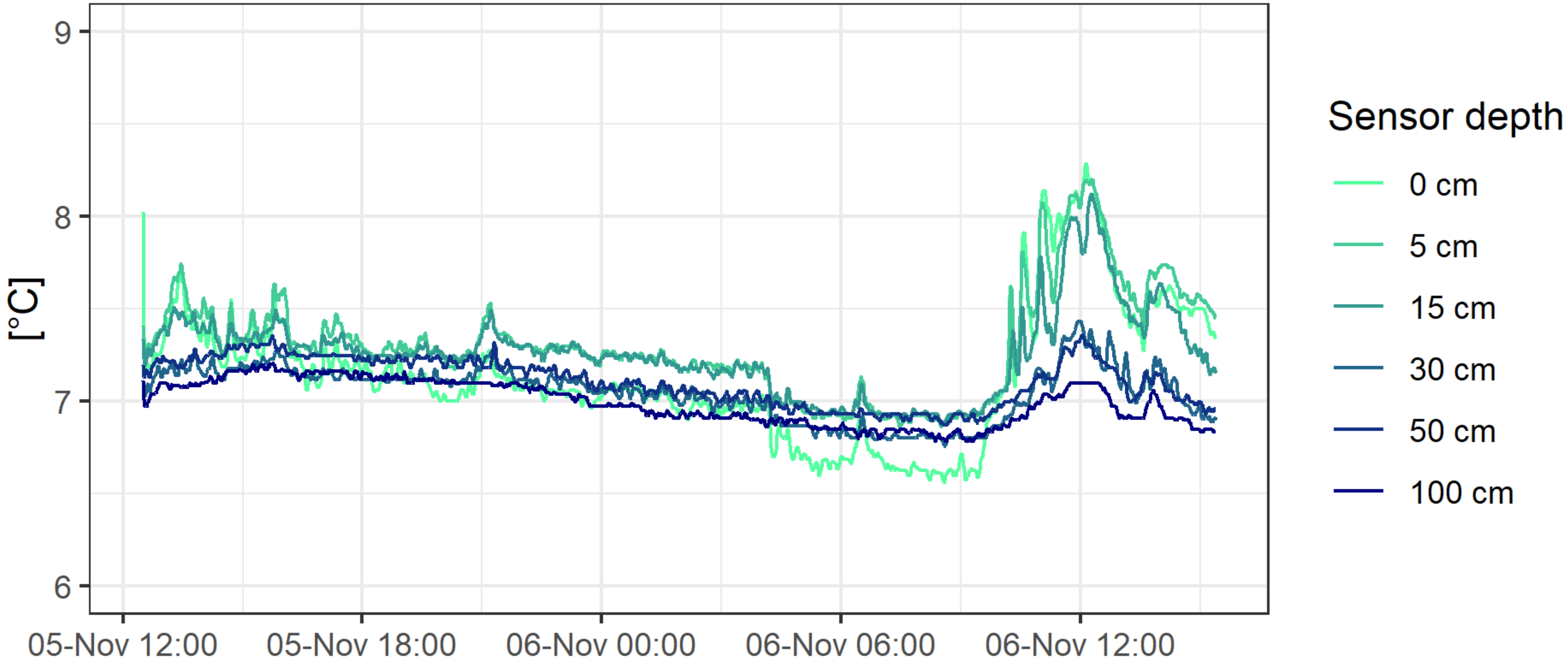

Figure S3.4: complete timeseries for SMeTD03 during field experiment

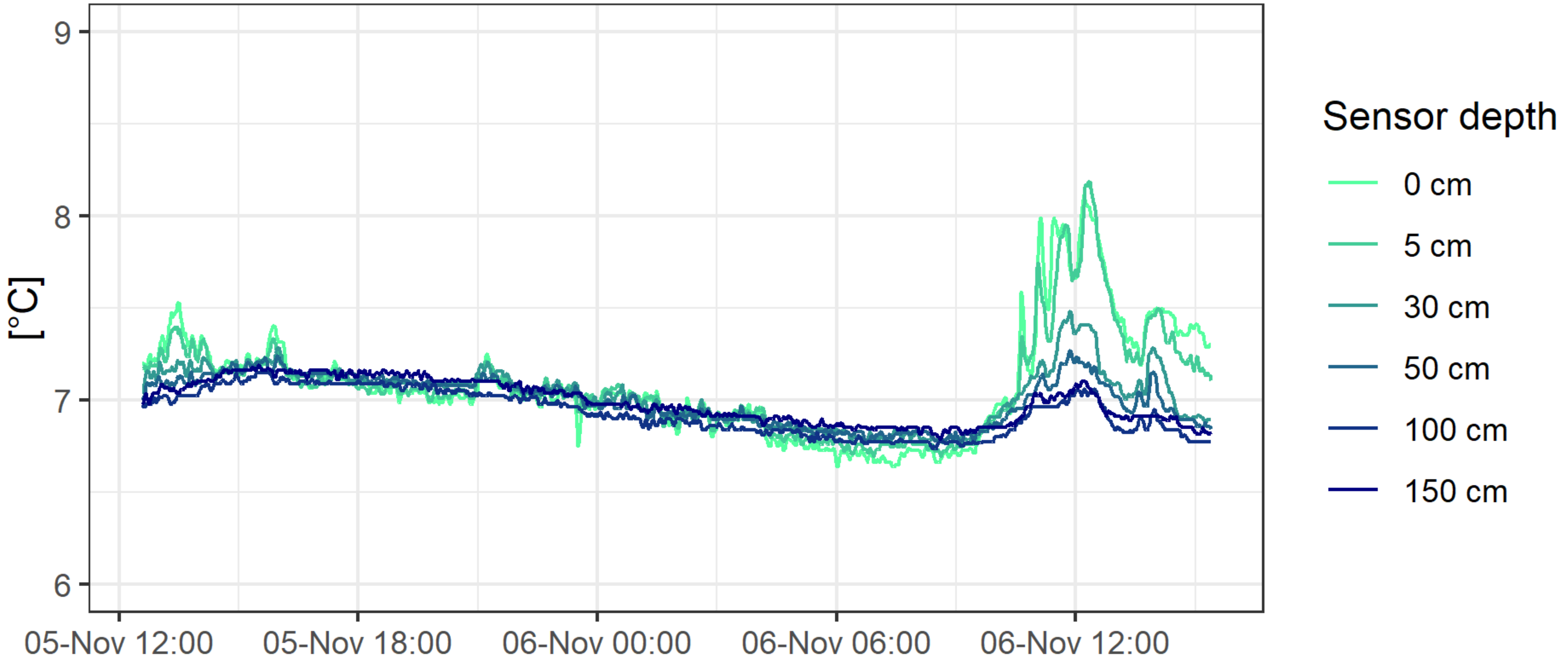

Figure S3.5: complete timeseries for SMeTD04 during field experiment

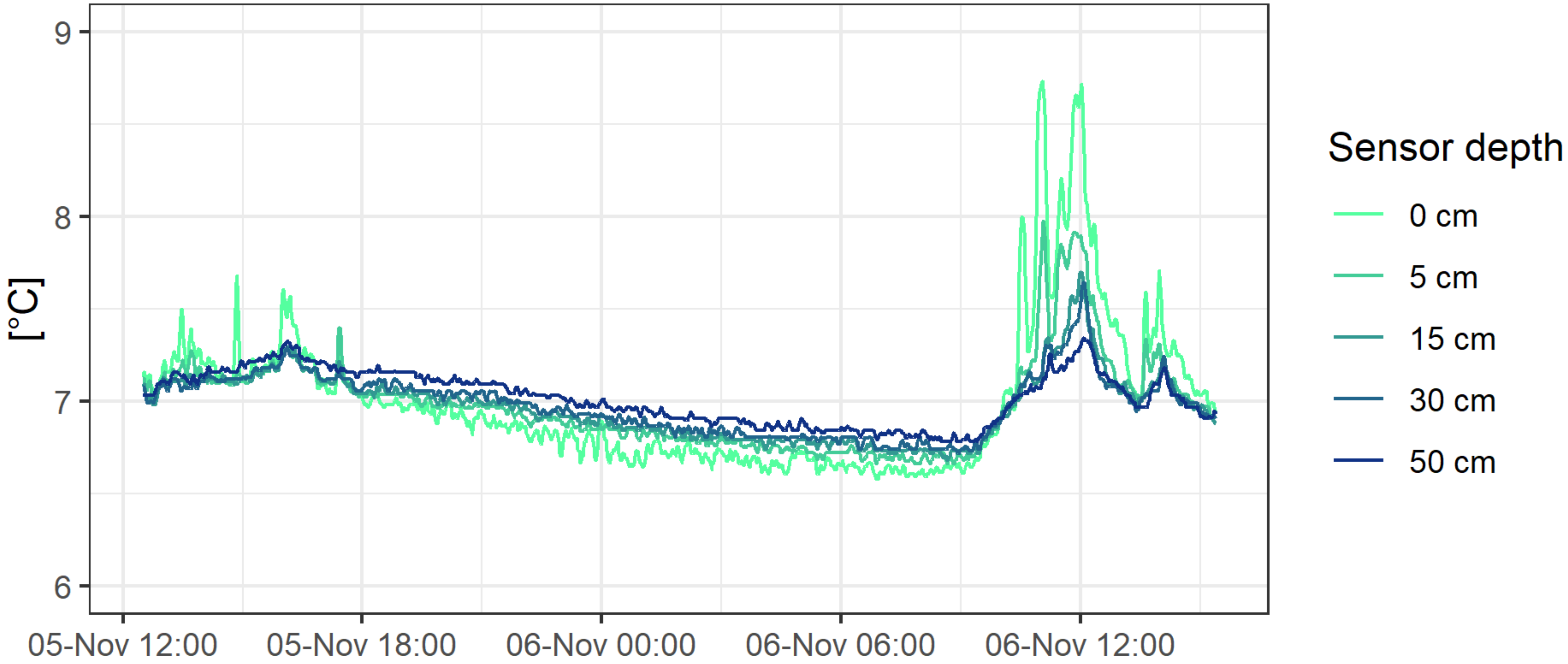

Figure S3.6: complete timeseries for SMeTD05 during field experiment

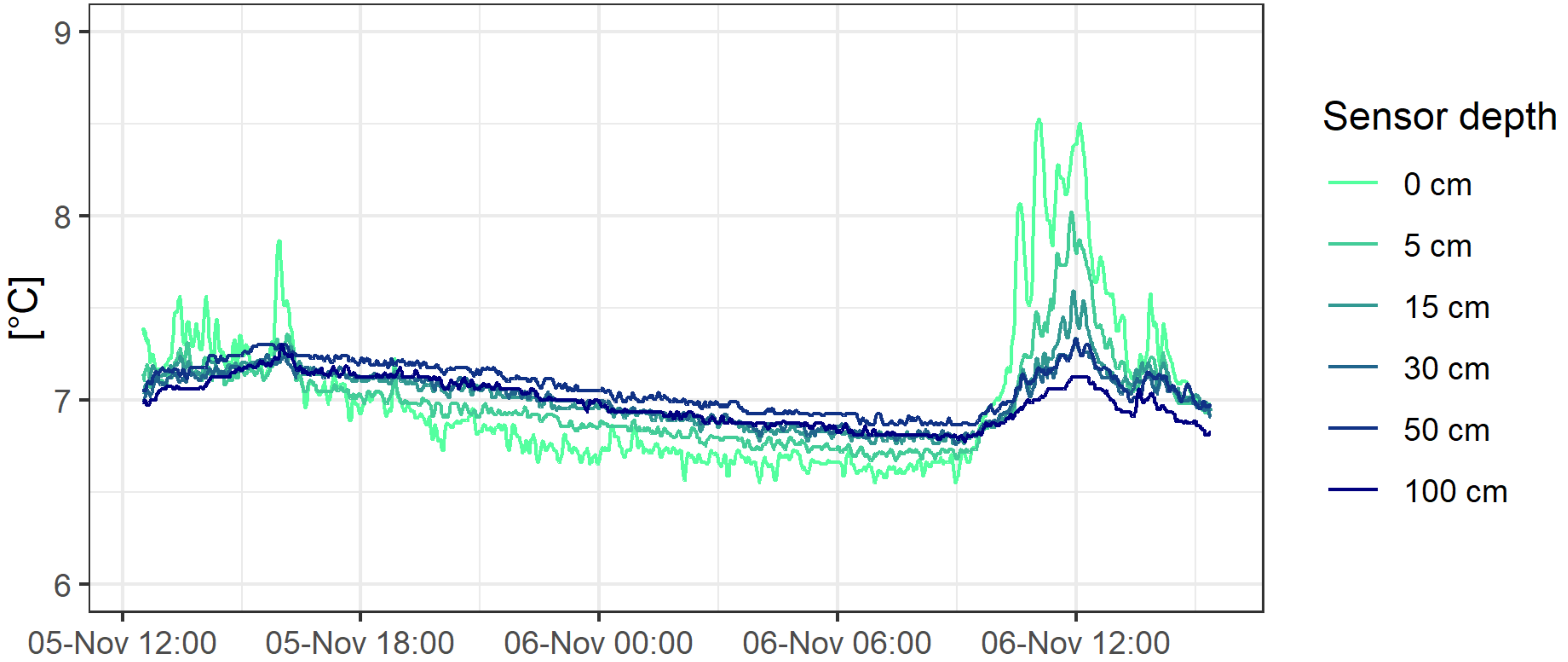

Figure S3.7: complete timeseries for SMeTD06 during field experiment

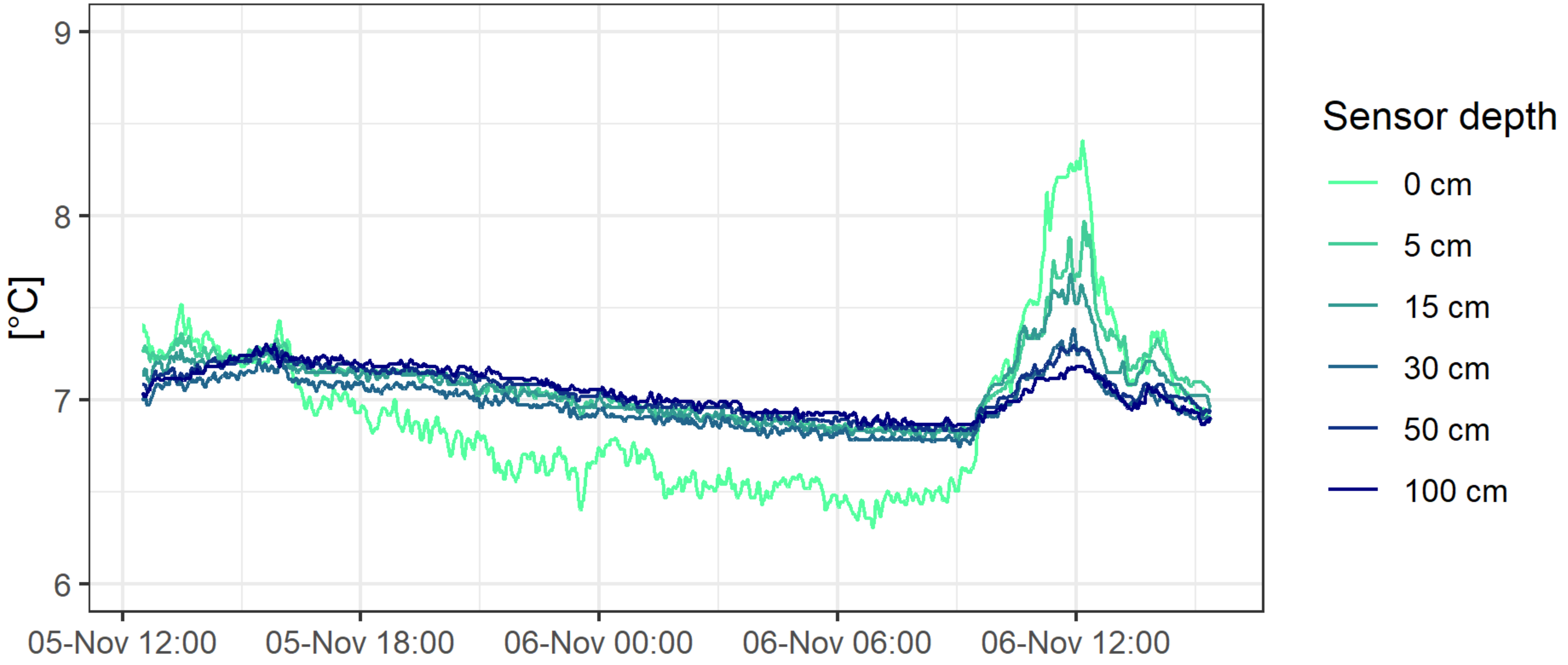

Figure S3.8: complete timeseries for SMeTD07 during field experiment

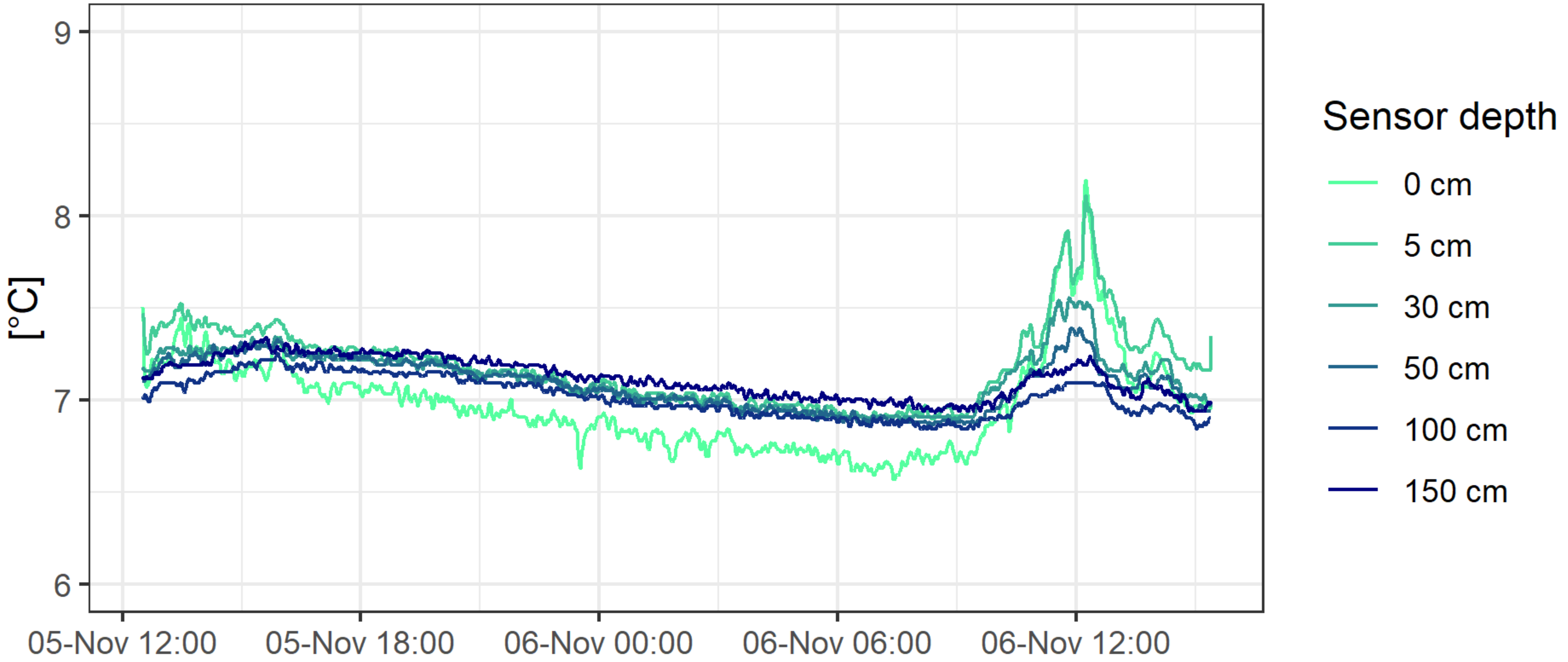

Figure S3.9: complete timeseries for SMeTD08 during field experiment

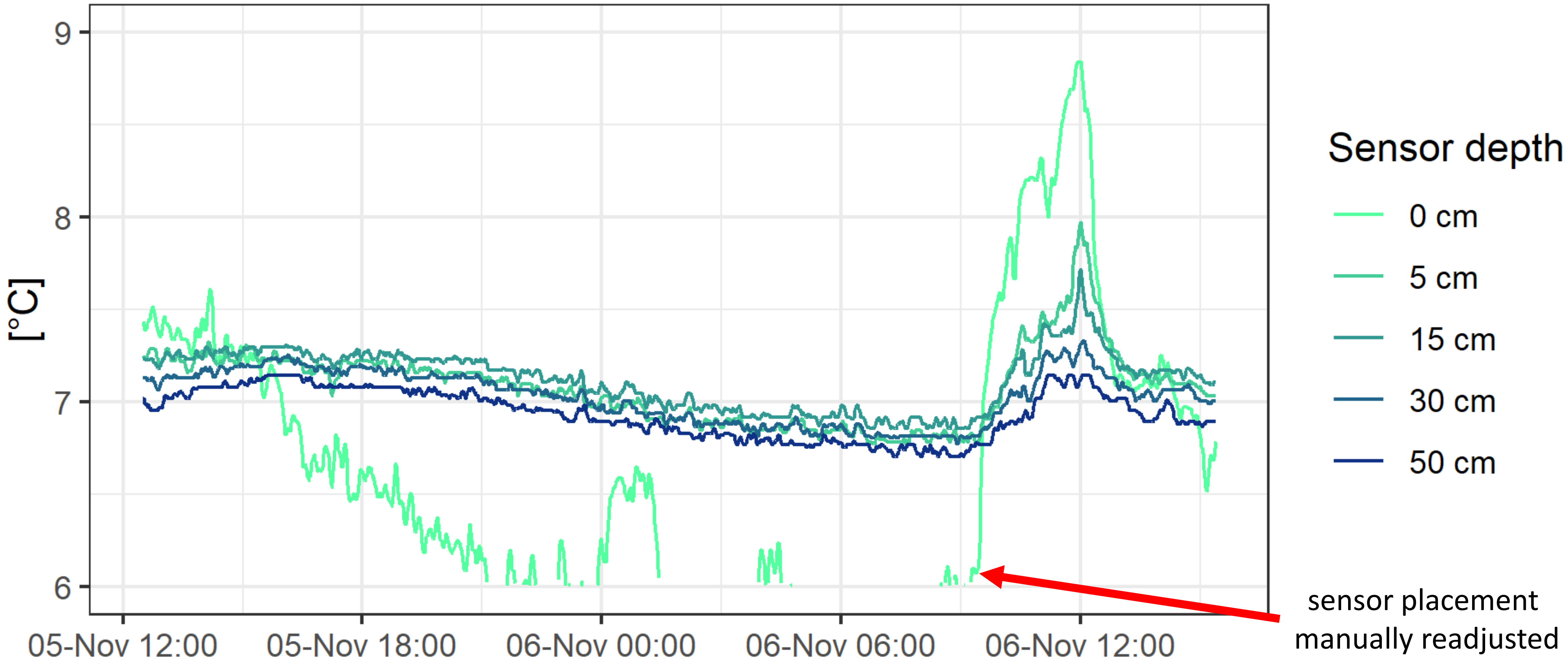

Figure S3.10: complete timeseries for SMeTD09 during field experiment

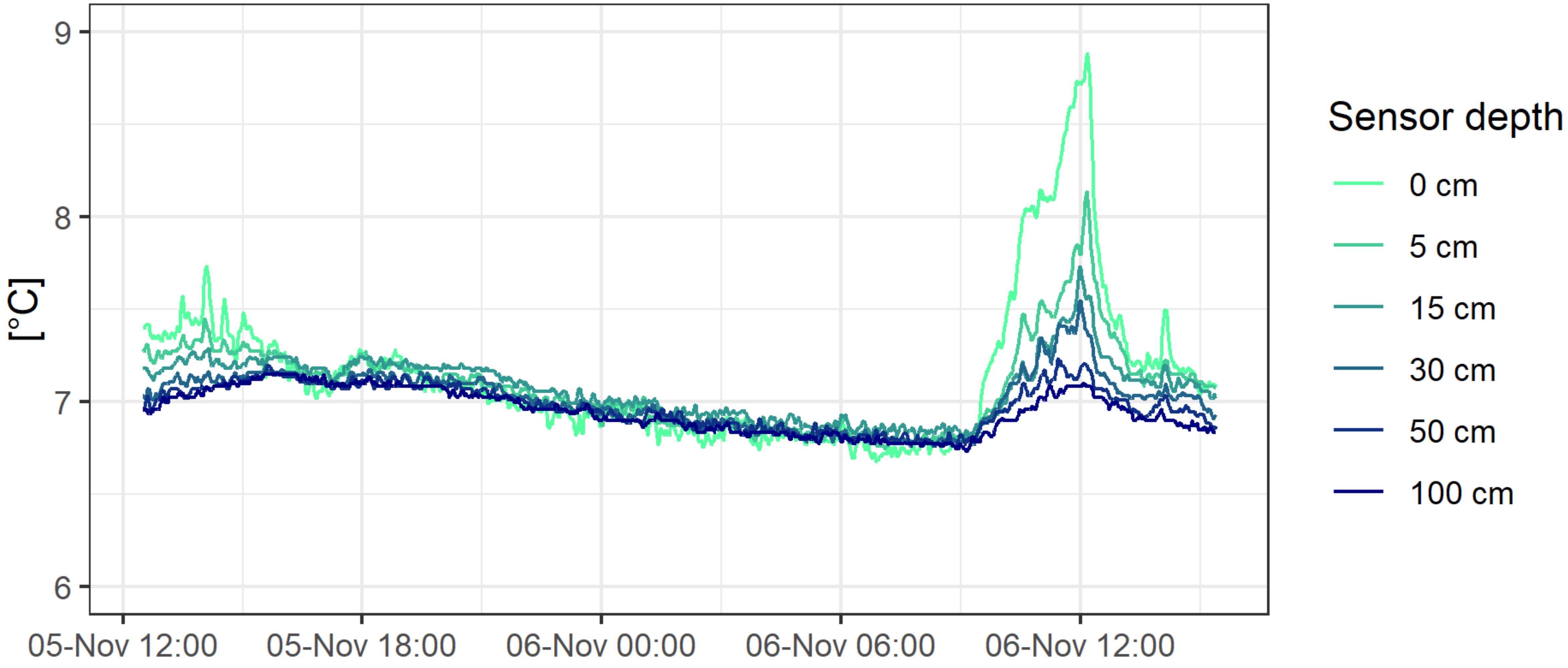

Figure S3.11: complete timeseries for SMeTD10 during field experiment
